# Artesunate induces ferroptosis in diffuse large B-cell lymphoma cells by targeting PRDX1 and PRDX2

**DOI:** 10.1038/s41419-025-07822-7

**Published:** 2025-07-11

**Authors:** Xiaohui Liu, Liyi Zeng, Jing Liu, Yulun Huang, Hua Yao, Jinman Zhong, Jiewen Tan, Xuejuan Gao, Dan Xiong, Langxia Liu

**Affiliations:** 1 MOE Key Laboratory of Tumor Molecular Biology and Key Laboratory of Functional Protein Research of Guangdong Higher Education Institutes, Institute of Life and Health Engineering, Jinan University, Guangzhou, China; 2 Department of Hematology, Shunde Hospital, Southern Medical University (The First People’s Hospital of Shunde), Foshan, Guangdong China

**Keywords:** Mechanism of action, Drug development, Drug regulation, Targeted therapies, Cell death

## Abstract

Diffuse large B-cell lymphoma (DLBCL), the most prevalent non-Hodgkin lymphoma (NHL), is characterized by rapid growth and an unfavorable prognosis. Artesunate (ART), a derivative of Artemisinin, is a widely recognized antimalarial drug that displays notable antitumor properties across diverse cancers. Our previous studies have demonstrated ART’s capacity to inhibit DLBCL progression *via* the induction of ferroptosis. However, its direct target molecules mediating this effect remained elusive. In this study, using small molecule (SM) pull-down combined with mass spectrometry analysis (LC–MS/MS), we have identified two peroxidases, PRDX1 and PRDX2, which play key roles in cellular antioxidant processes, as potential target proteins for ART in the treatment of DLBCL cells. Subsequently, we utilized cellular thermal shift assay (CETSA), fluorescence titration, circular dichroism (CD) spectroscopy, molecular docking, and pull-down assays to confirm that ART directly binds to PRDX1 and PRDX2. The Gly4 residue on PRDX1 and the Arg7 and Thr120 residues on PRDX2 are respectively responsible for ART binding. Knockdown of either PRDX1 or PRDX2 not only reproduced the ferroptosis-inducing effect of ART but also significantly attenuated the sensitivity of cells to ART. Furthermore, PRDX2 overexpression attenuated the reactive oxygen species (ROS) production and cytotoxicity in cells treated with ART. Consistently, ART selectively killed DLBCL cells, sparing normal cells thanks to their lower PRDX1 expression. In nude mice bearing U2932 xenografts, ART treatment inhibited significantly tumor growth and improved liver function without causing cardiac or hepatic toxicity, which was associated with an elevated level of ferroptosis and a significantly decreased peroxidase activity. Collectively, our findings elucidate the molecular mechanism by which ART induces ferroptosis through direct interaction with PRDX1 or PRDX2 and highlight these PRDXs as potential therapeutic targets for DLBCL.

## Introduction

Diffuse large B-cell lymphoma (DLBCL) represents a genetically and phenotypically heterogeneous malignant neoplasm of the lymphatic system, constituting the most prevalent subtype of non-Hodgkin lymphomas (NHLs), and accounting for approximately 50% of all NHL cases [1, 2]. The majority of patients with DLBCL respond favorably to the standard first-line treatment, R-CHOP (rituximab, cyclophosphamide, doxorubicin, vincristine, and prednisone). Nevertheless, a significant proportion, ranging from 30% to 40%, experience disease relapse or develop refractory DLBCL following initial treatment [3, 4]. Conventional salvage chemotherapy followed by high-dose therapy and autologous stem cell transplantation (ASCT) offers a viable treatment option for relapsed/refractory DLBCL. However, its efficacy remains moderate in achieving long-term remission, often limited by factors such as therapy resistance, comorbidities, or senility [5–7]. Therefore, there is an ongoing need for more effective and tolerable therapeutics for relapsed or refractory DLBCL patients. Our previous studies have shown that the development of DLBCL can be inhibited with artesunate (ART) treatment by inducing cell ferroptosis, a form of iron-dependent cell peroxidation death that differs from apoptosis, necrosis, and autophagy [8].

ART is a synthetic derivative of the traditional Chinese herbal medicine *Artemisia annua,* which has garnered significant attention in recent years for its potential anti-cancer properties, particularly in the treatment of various solid tumors. Originally developed as an anti-malarial drug, ART has been shown to exhibit a range of biological activities beyond its primary use, including the inhibition of cell proliferation [9], and the induction of cell death *via* apoptosis [10], ferroptosis [11], and autophagy [12]. Furthermore, ART has been reported to enhance the efficacy of conventional chemotherapeutic agents [10, 11], such as cisplatin [13], by exhibiting synergistic effects that lead to improved anti-tumor activity. This combinatorial therapy not only enhances the cytotoxic effects on cancer cells but also reduces the potential of drug resistance, a common challenge in cancer treatment.

While ART has shown promising anti-tumor potential across multiple cancer types, a comprehensive understanding of its molecular targets and mechanisms of action remains to be fully elucidated. Notably, little has been reported about the direct molecular targets of ART in cells. It is not until the last two years that there have been three publications possibly related to this subject. In 2023, S. Hill et al. combined computer modeling with UV-visible spectroscopy analysis to identify cytochrome c as a potential target protein of ART. They found that ART can induce apoptosis and abnormal mitochondrial membrane potential in pediatric acute myeloid leukemia (AML) cells that could be antagonized by methazolamide [14]. In 2024, Liu et al. uncovered that ART regulates the interplay between mitochondrial protease LONP1 and CYP11A1, a cytochrome P450 family member, affecting steroid hormone synthesis and metabolism, which holds promise for treating polycystic ovary syndrome (PCOS) [15]. More recently, Zhang et al. employed computational simulations and multiomics analyses to illustrate that ART acts by directly binding to myeloid differentiation factor 2 (MD2), presenting a novel therapeutic strategy for cardiac fibrosis [16]. We have previously demonstrated that ART induces ferroptosis and exhibits synergistic effects with doxorubicin in DLBCL cells [8]. In this study, we employed SM-pull down coupled with liquid chromatography tandem-mass spectrometry (LC–MS/MS) to identify two peroxidases, PRDX1 and PRDX2 (Peroxiredoxin 1 and 2) as the immediate protein targets of ART in DLBCL cells. A series of subsequent in vitro, in silico, and in vivo experiments have allowed us to confirm the direct interaction of ART with these two peroxidases, as well as its inhibitory effect on their enzymatic activity and the resulting functional consequences in DLBCL development.

## Results

### Identification of ART target proteins using SM-Pull down and LC–MS/MS

Our prior transcriptome sequencing analysis demonstrated that the ferroptosis pathway was significantly upregulated in U2932 cells following ART treatment. In this study, we first detected the degree of lipid peroxidation in DLBCL cells treated with ART. As shown in Fig. S1A, ART significantly increased the total ROS levels in germinal center B-cell-like (GCB) cells (Oci-Ly8) and activated B-cell-like (ABC) cells (Riva, U2932) using DCFH-DA fluorescent probe. Subsequently, the lipid peroxidation levels were detected using the C11-BODIPY 581/591 fluorescent probe in ART-treated DLBCL cells. The results showed that ART markedly induced lipid peroxidation in three DLBCL cells (Fig. S1B). Consistent with this, Malondialdehyde (MDA) levels, the end products of lipid peroxidation, were upregulated in three DLBCL cells with ART treatment (Fig. S1C). Furthermore, the protein levels of glutathione peroxidase GPX4, a marker of ferroptosis, was significantly down-regulated in ART-treated U2932 cells, whereas this effect was markedly blocked by the co-incubation with a ferroptosis inhibitor Fer-1, or an inhibitor of lipid peroxidation, liproxstatin-1 (Lip-1) (Fig. S1D), strongly supporting ferroptosis as the primary mechanism.

**Fig. 1:**
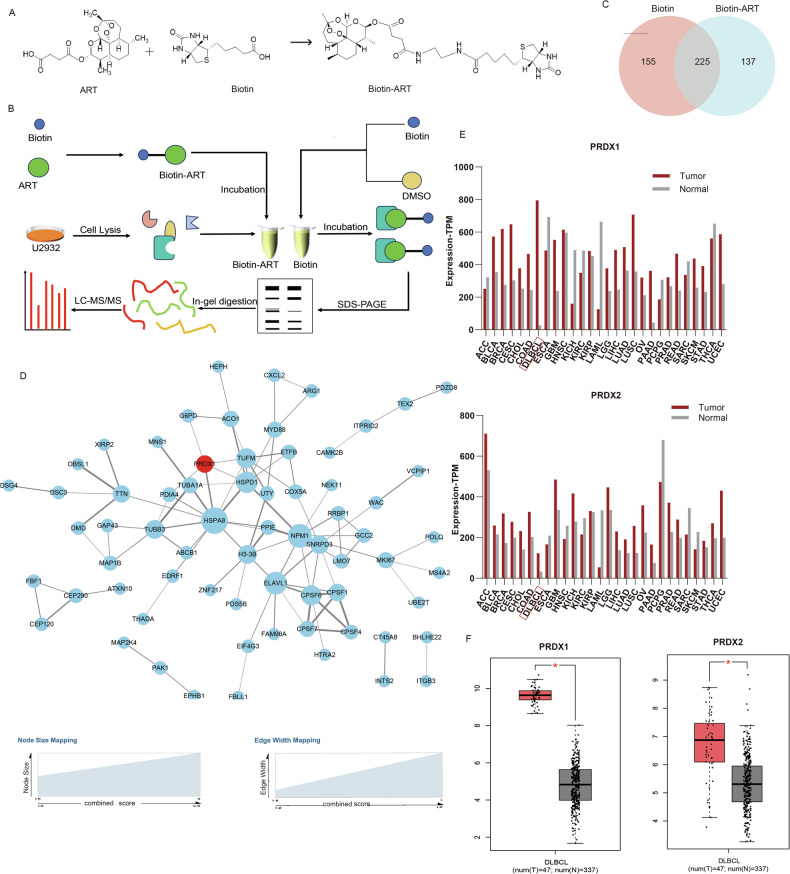
Identification of ART target proteins using SM-Pull down and LC–MS/MS. **A** Chemical structure of ART, Biotin, and Biotin-ART. **B** Overall workflow for SM-Pull down and LC–MS/MS profiling of potential ART targets. **C** The Venn diagram of the Biotin-ART group and the control group. **D** The unique proteins of the Biotin-ART group were analyzed by String. **E**, **F** TCGA online analysis (http://gepia.cancer-pku.cn/) of PRDX1 and PRDX2 expression in multiple tumors (**E**); Both genes are significantly overexpressed in DLBCL (**F**). The mean ± s.d. values for three biological replicates are shown. *p < 0.05.

To explore the ART interactome in DLBCL cells, the hydroxyl end of ART was bound to biotin by an amide bond to yield the Biotin-ART construct, whose structure was validated using high-resolution nuclear magnetic resonance (HNMR) (Fig. 1A and Fig. S2A). Both ART and Biotin-ART demonstrate concentration-dependent inhibition of cell viability, with increasing efficacy at higher concentrations (Fig. S2B). Furthermore, these cytotoxic effects can be rescued by a classical inhibitor of ferroptosis, Fer-1, or an inhibitor of lipid peroxidation, Lip-1, strongly supporting ferroptosis as a major process targeted by ART (Fig. S2C).

The interacting proteins of ART in DLBCL cells were isolated using small-molecule pull-down followed by LC–MS/MS (Fig. 1B). A total of 380 proteins were detected in the Biotin group and 362 proteins in the Biotin-ART group. Notably, 137 proteins were unique to the Biotin-ART group (Fig. 1C and Table S1). The interactions among these 137 unique proteins were analyzed using STRING, and the protein interaction network of ART was established, comprising 97 connections and 68 nodes. In this network, the protein PRDX1 has attracted our attention as being one of the core nodes (marked in red) (Fig. 1D). PRDX1 belongs to the family of Peroxiredoxins (PRDXs), which constitutes one of the most significant antioxidant systems in the body, critical for maintaining the cellular redox environment. It is involved in multiple ROS-dependent signaling pathways and plays a key role in the regulation of cell death [17–20]. Mammalian cells express six different PRDX isoforms (PRDX1-6). Both PRDX1- and PRDX2-containing peptide sequences were revealed by the MS data analysis in our study. Furthermore, analyses with TCGA database also revealed that, among the 28 cancer types investigated, both PRDX1 and PRDX2 proteins exhibited the most significant differential expression levels in DLBCL tumor (Fig. 1E, F), suggesting important roles of these two proteins in the development of DLBCL, and by that, the potential therapeutic targets of ART and other drugs.

### ART directly targets PRDX1 and PRDX2

**Fig. 2:**
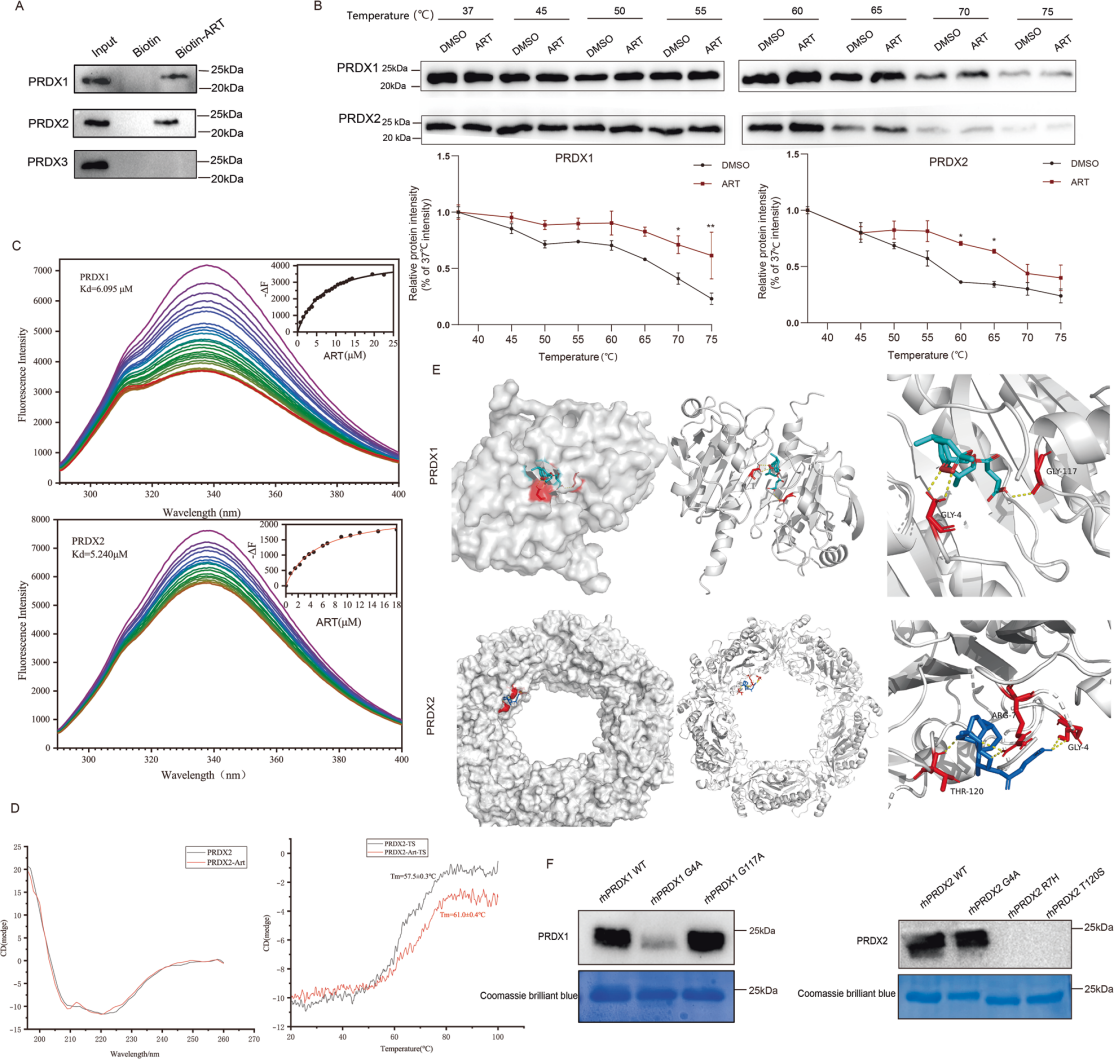
ART directly targets PRDX1 and PRDX2. **A** SM-Pull down and western blotting assay to verify that ART directly targets PRDX1 and PRDX2 proteins. The experiments were performed in three independent biological replicates. **B** Cellular thermal shift assay (CETSA)-western blotting to test the interactions of ART with PRDX1 or PRDX2 proteins in U2932 cells. Data are presented as mean ± s.e.m. from three independent experiments. *: *p* < 0.05, **: *p* < 0.01. **C** The binding constants of PRDX1/PRDX2 with ART, measured by fluorescence titration, were 6.095/5.240 μM (by Hill equation). **D** Circular dichroism (CD) spectroscopy was used to monitor the changes of secondary structure upon rhPRDX2 binding to ART. **E** Molecular docking results of ART with PRDX1 and PRDX2 proteins. **F** The recombinant PRDX1 and PRDX2 mutant proteins were subjected to Biotin-ART and pulled down with streptavidin beads.

We then repeated the pull-down experiment to confirm the direct interaction between ART and PRDX1 or PRDX2. The results of western blotting following the pull-down assays show that Biotin-ART specifically interacted with PRDX1 and PRDX2, but not with PRDX3, which is another PRDX family member lacking the MS-identified peptide sequences (Fig. 2A). We then performed CETSA (Cellular Thermal Shift Assay) to further validate these interactions (Fig. 2B). The CETSA assay is based on the principle that ligand binding can modify protein thermal stability, which can be reflected by the change of protein amount under varying temperature and visualized using western blotting. Protein extracts from U2932 cells were treated with ART (100 μM) or DMSO, and subjected to CETSA heat pulse followed by soluble protein extraction and quantification. Interestingly, both PRDX1 and PRDX2 displayed significant thermal stabilization in the presence of ART. In addition, a fluorescence spectrometry assay was also carried out with the rhPRDX1 and rhPRDX2 proteins to investigate their dynamic properties in the presence or absence of ART. The results showed a remarkable binding of ART with both rhPRDX1 and rhPRDX2, with Kd values of 6.095 and 5.240 μM, respectively (Fig. 2C). Circular dichroism (CD) spectroscopy was used to monitor the alteration of secondary structure in rhPRDXs upon their binding to ART. The contents of the α-helix, β-sheet, β-turns, and random coils calculated by the CDNN software package (Bio-Logic) were listed in Table 1. The CD spectra of rhPRDX2 in the absence or presence of ART at different temperatures were shown in Fig. 2D. A decrease in α-helix and an increase in β-sheet content were observed in rhPRDX2 upon its binding with ART, accompanied by a rise of the Tm of the protein from 57 °C to 61 °C. Curiously, no significant impact on the secondary structure of rhPRDX1 has been detected in our experiments (data not shown). This suggests that nuances might exist at the sub-molecular level between PRDX1 and PRDX2 in their binding with ART.

**Table 1:** Secondary structural analyses based on circular dichroism spectral measurements.

sample	α-helix	β-sheet	β-turns	random coils
PRDX2	23.2%	27.8%	18.7%	42.40%
PRDX2-Art	22.1%	29.4%	19.00%	42.80%

CD analysis of secondary structures of PRDX2 and its interactions with ART, calculated by CDPro software.

To further investigate the molecular interaction of ART with PRDX1/2 at a more precise level, we sought to identify the amino acid residues on PRDX1/2 that are directly involved in ART binding. Molecular docking analyses were performed between ART and PRDX1 or PRDX2. The results suggested that ART could be successfully docked in the dimer interface of PRDX1 dimer through its direct interaction with the Gly4 and Gly117 residues of the latter (Fig. 2E, upper panels). Quite differently, the binding pocket of ART on PRDX2 is located in the proximity of the molecular junctions of PRDX2 decamer, involving the Gly4, Arg7, and Thr120 residues of PRDX2 (Fig. 2E, lower panels). In order to confirm these findings, a series of single-point mutants, namely PRDX1-G4A, PRDX1-G117A, PRDX2-G4A, PRDX2-R7H, and PRDX2-T120S, were generated by site-specific mutagenesis. These recombinant PRDX1 and PRDX2 mutant proteins were first subjected to Biotin-ART pull-down assays. The results indicated that ART-PRDX1 binding was mediated by the Gly4 residue on PRDX1 (Fig. 2F), whereas the Arg7 and Thr120 residues of PRDX2 were responsible for its binding to ART.

### ART inhibits the antioxidant activities of PRDX1 and PRDX2 without affecting their expression level

**Fig. 3:**
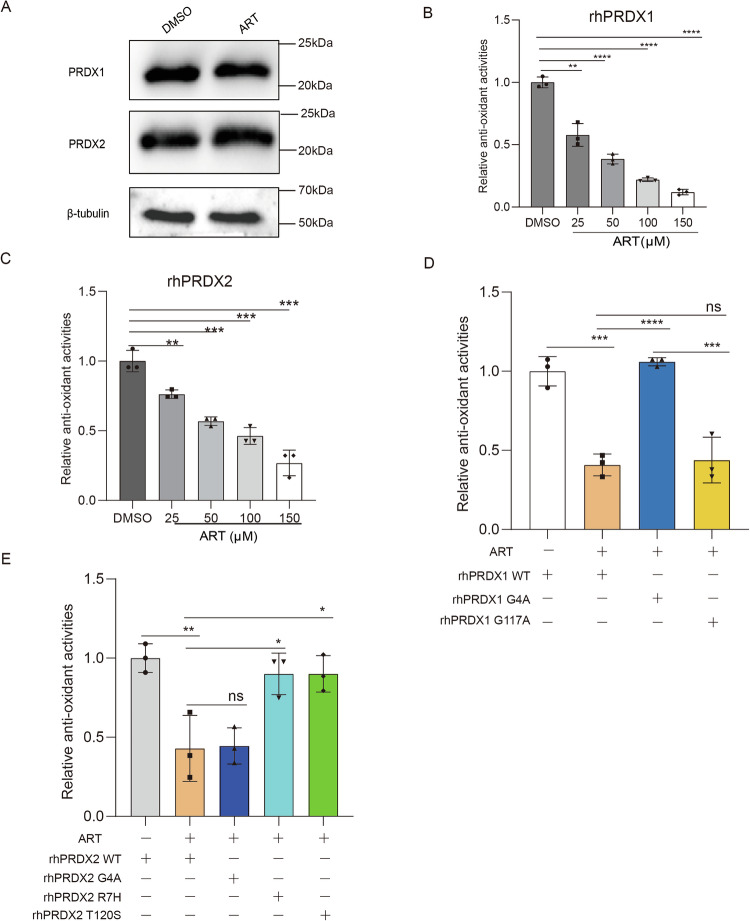
ART inhibits the antioxidant activities without affecting the expression levels of PRDX1 and PRDX2. **A** The expression levels of PRDX1 and PRDX2 proteins in U2932 cells treated with DMSO and ART for 24 h were detected by western blotting assay. rhPRDXs (20 μM) was co-incubated with ART of different concentrations for 30 min, 10 μL H_2_O_2_ was added (final 100 μM), and then residual H_2_O_2_ level was detected by hydrogen peroxide detection kit, and enzyme activities of PRDX1 (**B**), PRDX2 (**C**) and their recombinant mutant proteins (**D**, **E**) were determined. Data are presented as mean ± s.d. from three independent experiments. *: *p* < 0.05, **: *p* < 0.01, ***: *p* < 0.001, ****: *p* < 0.0001, determined by one-way ANOVA.

To determine the effect of ART binding on the PRDXs’ function, we first measured the protein level of PRDX1 and PRDX2 after ART treatment of U2932 cells. The results showed that ART had no effect on the expression level of PRDX1 and PRDX2 (Fig. 3A), excluding the possibility that ART might alter the PRDXs’ function by regulating their expression level. We further explored whether ART affected the antioxidant activity of recombinant human PRDXs (rhPRDX1 and rhPRDX2) using an in vitro H_2_O_2_ reduction assay. The results showed that ART suppressed the peroxidase activity of rhPRDX1 and rhPRDX2 in a dose-dependent manner (Fig. 3B, C). Interestingly, ART had no effect on the mutant forms of rhPRDX1 (G4A) and rhPRDX2 (R7H and T120S), which had lost their capability to interact with it, while those (rhPRDX1-G117A and rhPRDX2-G4A) still capable of binding remained sensitive to its inhibition (Fig. 3D, E). These results suggest that ART can directly bind to PRDX1 and PRDX2 proteins and inhibit their enzymatic activities.

### Knockdown of PRDX1 or PRDX2 proteins stimulated ROS production and induced ferroptosis

**Fig. 4:**
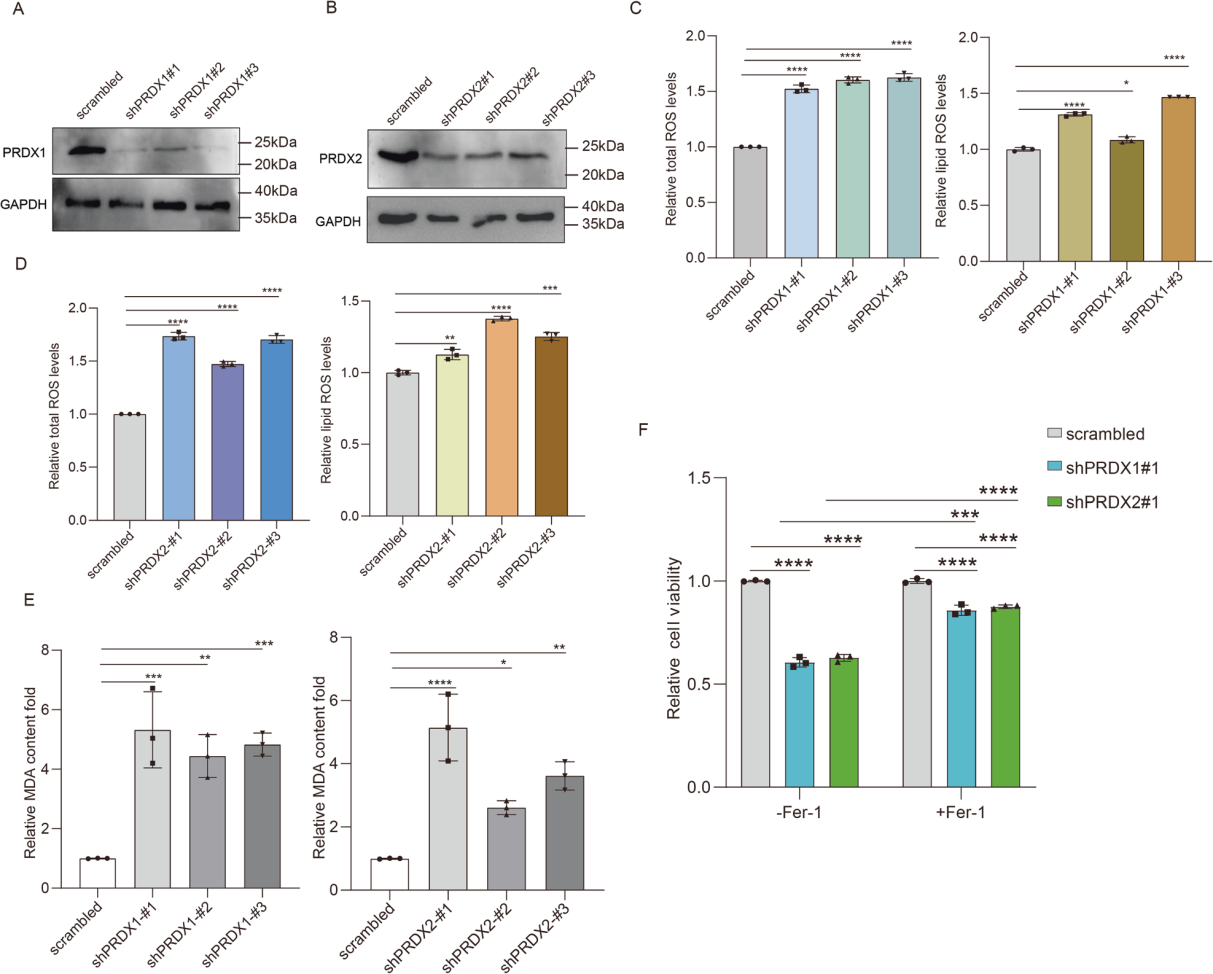
Knockdown of PRDX1 or PRDX2 proteins aggravates ROS signaling and induces ferroptosis. Western blotting assay verified the effect of stable knockdown of PRDX1 (**A**) and PRDX2 (**B**) in 293 cells. PRDX1 knockdown (**C**) or PRDX2 knockdown (**D**), or control 293 cells were labeled with a 10 μM DCFH-DA or 10 μM C11-BODIPY fluorescent probe, respectively, and total ROS or lipid ROS levels were detected by flow cytometry. **E** Detection of cellular MDA levels in PRDX1 or PRDX2 knockdown 293 cells. **F** The cell viability of 293 cells with stable knockdown of PRDX1 and PRDX2 was measured by CCK-8 assay, and after 24 h treatment with Fer-1 (50 μM), a ferroptosis inhibitor. Data are presented as mean ± s.d. from three independent experiments. *: *p* < 0.05, **: *p* < 0.01, ***: *p* < 0.001, ****: *p* < 0.0001, determined by one-way or two-way ANOVA.

To functionally validate the PRDX1 and PRDX2 proteins as the target proteins of ART in the induction of ferroptosis with antiproliferative consequences, three lentiviral expressing shRNA for each of these two proteins were designed and synthesized (Table S2). The efficacy of these shRNAs was evaluated by transfection of human embryonic kidney 293 cells with the corresponding shRNAs, followed by western blotting analysis of the indicated protein levels (Fig. 4A, B). Subsequently, DCFH-DA or C11-BODIPY probes were used to detect the level of total or lipid ROS in the transfected cells. The results showed that PRDX1 or PRDX2 knockdown resulted in significant accumulation of total ROS and lipid ROS (Fig. 4C, D). Furthermore, knockdown of PRDX1 or PRDX2 proteins led to elevated MDA levels (Fig. 4E). Finally, PRDX1 or PRDX2 knockdown significantly inhibited cell viability, as indicated by the results of CCK-8 assay, and more importantly, this could be reversed by the ferroptosis inhibitor Fer-1 (Fig. 4F). Collectively, these results indicated that PRDX1 or PRDX2 knockdown enhanced the ROS production and thereby regulated the ferroptosis.

### PRDX1 and PRDX2 proteins are the key target proteins of ART in ROS production, leading to cell death

**Fig. 5:**
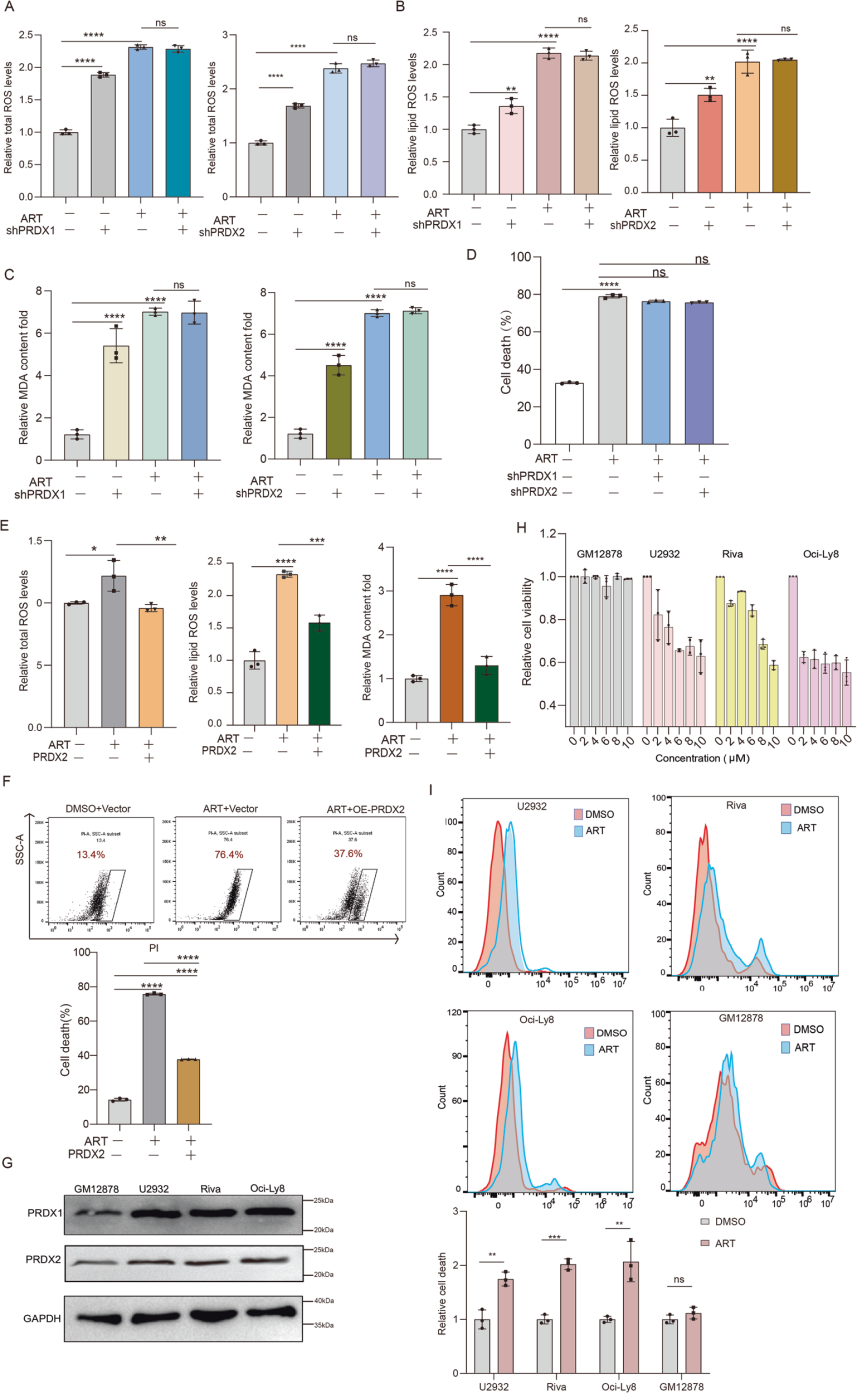
PRDX1 and PRDX2 proteins are the key target proteins of ART-induced ferroptosis. The total ROS (DCFH-DA) (**A**), the lipid ROS levels (**B**), the MDA levels (**C**), and cell death (**D**) measurements in shRNA-mediated PRDX1 or PRDX2 knockdown, or non-target scrambled 293 cells treated with or without ART (100 μM). 293 cells pretreated with 100 μM ART were transfected with a plasmid expressing flag-PRDX2 fusion protein; the total ROS, lipid ROS, and MDA levels (**E**) and cell death (**F**) were then detected. **G** The expression levels of PRDX1 and PRDX2 protein in human normal B lymphocytes and three cell lines of DLBCL were detected by western blotting assay. **H** The cell viability of GM12878, U2932, Riva, and Oci-Ly8 cells treated with ART under the indicated gradient concentration was determined by CCK-8 assay. **I** The proportion of dead cells in GM12878, U2932, Riva, and Oci-Ly8 cells after ART treatment for 24 h was determined by the propidium iodide exclusion assay. Data are presented as mean ± s.d. from three independent experiments. *: p < 0.05, **: *p* < 0.01, ***: *p* < 0.001, ****: *p* < 0.0001, determined by one-way or two-way ANOVA.

The above results indicated that ART-induced cell death through enhancing ROS production. We then proceeded to elucidate whether it was indeed by the inhibition of PRDX1 and PRDX2 that ART exerted such activities. PRDX1 or PRDX2 was knocked down in the cells, and the above-mentioned effects of ART were re-examined. It turned out that ART treatment did not significantly increase total ROS (Fig. 5A), lipid ROS (Fig. 5B), or MDA accumulation (Fig. 5C), and showed no additional effect on cell death when PRDX1 or PRDX2 was knocked down (Fig. 5D). Conversely, overexpression of PRDX2 fully rescued the increased ROS levels and cell death in ART-treating cells (Fig. 5E, F). Due to the repeated failures of exogenous PRDX1 overexpression in the cells, we were unable to perform similar experiments for PRDX1, but results similar to those of PRDX2 could be expected.

To further investigate the role of PRDX1 and PRDX2 on the susceptibility of cells to ART, we analyzed their expression levels in normal human B lymphocytes (GM12878) and three DLBCL cell lines (U2932, Riva, and Oci-Ly8). The results showed that both PRDX1 and PRDX2 were highly expressed across all DLBCL strains as compared to normal B lymphocytes (Fig. 5G). Subsequently, we conducted a CCK-8 assay and found that when treated with ART of increasing concentration, the cell viability of the three DLBCL cell lines significantly decreased, whereas no obvious impact could be observed in normal B lymphocytes (Fig. 5H). In addition, the propidium iodide exclusion assay showed a significant increase in the proportion of dead cells within the ART-treated DLBCL cohort compared to untreated controls, while normal B lymphocytes remained unaffected (Fig. 5I). These results together suggested that ART aggravated ROS production and induced ferroptosis through PRDX1 and PRDX2, and that ART selectively killed DLBCL cells rather than normal cells probably thanks to the elevated levels of peroxiredoxins in the malignant cells.

### ART inhibits DLBCL tumor growth and improves liver function without causing cardiac or hepatic toxicity in vivo

**Fig. 6:**
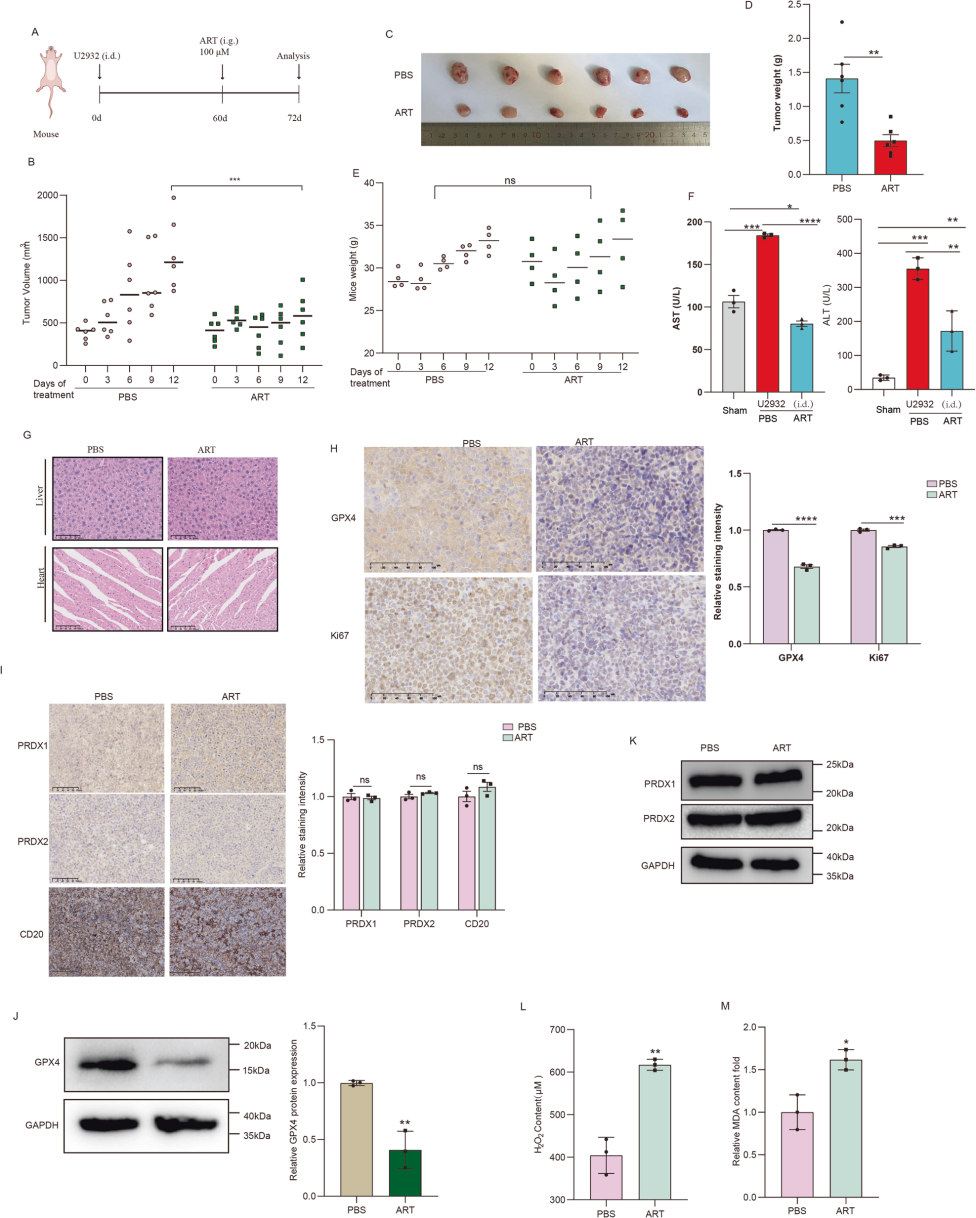
ART inhibits DLBCL tumor growth and improves liver function without causing cardiac or hepatic toxicity in vivo. **A** The schematic figure showing 6-week-old male nude mice subcutaneously injected with U2932 cells to establish DLBCL tumor models and then receiving intragastric administration of PBS or ART every 3 days (*N* = 6). Thereafter, the mice underwent the following assessments: measurement of tumor growth curve (**B**), tumor size (**C**), tumor weight (**D**), mice weight (**E**), ALT and AST in blood (**F**), H&E staining of cardiac and hepatic tissues (**G**), immunohistochemical analyses of tumor tissues (**H**, **I**), protein expression levels in tumor tissues (**J**, **K**), the levels of H_2_O_2_ in tumor tissues (**L**), and MDA levels (**M**). Data are presented as mean ± s.e.m. *p < 0.05, determined by two-way ANOVA.

To further investigate the effect of ART on DLBCL, we established a murine model *via* subcutaneous injection of U2932 cells into male BALB/c-Nude mice. Following successful tumor induction, mice were subjected to intragastric administration of PBS or ART every three days. Tumor dimensions and mouse body weights were meticulously monitored and recorded (Fig. 6A). Our findings demonstrated a substantial reduction in tumor volume and weight within the ART-treated cohort compared to controls (Fig. 6B–D), with no significant difference in body weight between groups (Fig. 6E). Subsequently, serum from the mice was extracted by means of orbital blood collection for the detection of liver function (ALT, AST), and the results showed that the liver function was significantly improved post-ART intervention (Fig. 6F). Histopathological examination *via* H&E staining of cardiac and hepatic tissues revealed no treatment-associated pathologies (Fig. 6G). Immunohistochemical (IHC) analyses indicated a marked decrease in GPX4, a key ferroptosis marker, following ART exposure, alongside a significant suppression of Ki67, indicative of reduced proliferation in DLBCL (Fig. 6H). There was no significant change in the protein expression of PRDX1, PRDX2 and CD20 (a DLBCL tumor cell marker) in either the control group or the ART group (Fig. 6I). Western blotting from the tumor tissue corroborated these observations, showing diminished GPX4 levels without alterations in PRDX1 and PRDX2 expressions (Fig. 6J, K). Furthermore, an H_2_O_2_ reduction assay with indicated tumor tissues confirmed the peroxidase activity inhibition under ART treatment (Fig. 6L). Meanwhile, the ART-treated group showed significantly elevated MDA levels (Fig. 6M), demonstrating enhanced lipid peroxidation in vivo. Collectively, these data confirmed that ART inhibits DLBCL progression, ameliorates liver function, and does not induce cardiac or hepatic toxicity in vivo*.*

## Discussion

Artesunate, a water-soluble hemisuccinate derivative of artemisinin with a double oxygen bridge structure [21], has shown great promise in a variety of applications with minimum side effects, such as treating malaria, inflammation, tumors, and immunomodulatory [15]. However, the direct target proteins mediating its actions in various pathological contexts and tissue specificities are poorly acknowledged. The exploration of such target proteins has just begun, as manifested by the three reports in the literature during the past two years, each characterized by different scientific backgrounds, methodology choices, and research outcomes [14–16]. Notably, the specific direct target proteins mediating ART’s therapeutic intervention in DLBCL remained unidentified prior to our research.

Our investigations verified ART’s capacity to activate ferroptosis pathways, suppress the proliferative capacity of both ABC subtype (U2932, Riva) and GCB subtype (Oci-Ly8) DLBCL cells, and induce ROS accumulation and lipid peroxide elevation (Fig. S1A–D). Although ART has been reported to promote other modes of cell death, such as apoptosis and autophagy [14, 22]. Our previous RNA-seq data showed that ferroptosis-related genes were top-ranked (ranked 3rd), while apoptosis/autophagy pathways were much less enriched (ranked 13th and >30th, respectively) [8]. Flow cytometric analysis in this study revealed that ART treatment induced a modest but statistically significant increase in apoptosis in U2932 cells compared to controls, though this effect remained substantially weaker than that induced by the classical apoptosis-inducing drug doxorubicin (DOX) (Fig. S1E). This in vitro observation was corroborated by in vivo studies showing moderate pro-apoptotic effects: both TUNEL staining and cleaved caspase-3 immunofluorescence demonstrated elevated apoptotic activity in ART-treated tumors (Fig. S3). While these findings confirm ART’s capacity to induce apoptotic cell death, its relatively weak pro-apoptotic activity compared to established inducers, combined with the well-documented susceptibility of DLBCL cells to ferroptosis [23], led us to focus our mechanistic investigation primarily on the ferroptosis pathway and its associated molecular regulators.

In this study, we pursued our investigation by identifying PRDX1 and PRDX2 as the direct target proteins of ART, which mediated its ferroptosis-inducing and antiproliferative functions in DLBCL. The results of our in vitro experiments with cultured cells and in vivo experiments using the nude mice bearing U2932 xenografts were corroborated by the analyses of clinical samples using TCGA public datasets, which demonstrated drastic overexpression of both PRDX1 and PRDX2 in DLBCL tissues of the patients.

Peroxiredoxins (PRDXs) constitute a crucial antioxidant system in mammalian cells, significantly modulating the cellular redox balance. Mammalian cells express six PRDX isoforms (PRDX1-6), with PRDX1, PRDX2, PRDX3, and PRDX4 categorized as typical 2-cysteine (2-Cys) PRDXs [24–26]. Despite variations in their cellular redox regulatory functions, these enzymes collectively catalyze the reduction of H_2_O_2_, organic hydroperoxide, featuring a conserved thioredoxin CxxC motif for electron donation [27–29]. Peptides bearing the same sequence commonly shared by PRDX1 and PRDX2 have been identified by the mass spectrometry analysis following Biotin-ART pull down in our study, suggesting that both peroxidases could be targeting proteins of ART.

Peroxiredoxin 1 (PRDX1) is a primarily cytoplasmic protein and can be activated *via* diverse mechanisms under oxidative conditions [26, 30]. Under stress conditions, PRDX1 can also serve as a molecular chaperone [31]. Beyond its roles as a peroxidase and molecular chaperone, PRDX1 enhances natural killer cell cytotoxicity and inhibits oncogenic proteins such as c-Myc and c-Abl [32, 33]. Recent studies have reported increased expression of PRDX1 in several human cancers, including breast, esophageal, lung, and prostate cancers [17, 34–36]. In our research, knockdown of PRDX1 resulted in an accumulation of the total and lipid ROS, coupled with increased levels of lipid peroxidation. Furthermore, the reduction in cell viability associated with PRDX1 knockdown was mitigated by ferroptosis inhibitors. These findings align with the literature indicating that PRDX1 is involved in various ROS-dependent signaling pathways and plays a pivotal role in regulating cell death [18, 37]. Interestingly, we showed in this study that DLBCL cell lines with elevated PRDX1 expression also exhibit heightened susceptibility to ART treatment. Together with the demonstration of ART-PRDX1 interaction, this observation underscores for the first time the potential of PRDX1 as a therapeutic target for DLBCL.

Peroxiredoxin 2 (PRDX2) is localized in the nucleus, cytoplasm, and cell membrane, and can form decameric structures in the cellular environment, thereby maintaining a high reaction rate with H_2_O_2_ [38–40]. PRDX2 expression is elevated in several human solid tumors such as lung adenocarcinoma, gastric cancer, and colorectal cancer [41–43]. In lung adenocarcinoma, PRDX2 levels correlate with both overall survival (OS) and disease-free survival (DFS) [25, 42]. For gastric cancer, higher PRDX2 expression is linked to advanced TNM stages, lymph node metastasis, and a poorer prognosis [43]. In our study, PRDX2 knockdown also activated ROS-dependent signaling pathways and induced ferroptosis, as observed for PRDX1.

Given that PRDX1 and PRDX2 are the closest homologs in the PRDX family and both present similar H_2_O_2_ reduction activity in the cell, one of them might therefore exert a compensatory effect in case of functional default of the other. In this regard, ART may turn out to be an advantageous drug owing to its capability to target both of these PRDXs. This is all the more interesting in the sense that our studies combining in silico molecular docking and site-specific mutagenesis have shown that different regions and amino acid residues in PRDX1 and PRDX2 are directly involved in the binding with ART, and within different polymeric conformations of the two proteins. In the portrayed molecular model, while PRDX1 formed a homodimer and was in association with the ART molecule at the dimer interface involving the Gly4 residue, PRDX2 was presented to be a decamer and associated with ART through its Arg7 and Thr120 residues at the proximity of the decamer interface.

The construction of PRDX1 and PRDX2 knockdown or overexpressed cell lines, combined with cell phenotype monitoring, has demonstrated that the anti-cancer activity of ART depends on these two proteins, further validating the physical and functional interaction of ART with peroxidases and elucidating its key role in ferroptosis. Additionally, we also demonstrated that ART significantly inhibited the proliferation of DLBCL by suppressing the antioxidant levels in an animal model, with no significant abnormalities or organ damage observed in the heart and liver, suggesting that ART is an effective and safe small-molecule drug for the treatment of refractory or relapsed DLBCL. It is worth noting that after ART treatment, the growth of tumors is inhibited without a decrease in CD20 expression in vivo, which may be due to several reasons. Firstly, ART may primarily act on downstream signaling pathways of CD20, thereby inhibiting tumor cell proliferation and survival without affecting CD20 expression itself. Secondly, tumor cells might activate compensatory mechanisms to maintain CD20 expression despite ART’s anti-tumor effects. Further studies are needed to clarify these mechanisms.

The identification of Gly4 in PRDX1 and Arg7 and Thr120 in PRDX2 as the binding sites of these two proteins to ART has brought our study to a sub-molecular level. This finding provides not only insight into the structural basis of the interactions, but also further evidence for the confirmation of the ART-PRDXs interactions and their functional significance.

Multiple reports related ART’s anti-malarial activity to ROS induction within *plasmodium falciparum* [44]. Furthermore, it has been shown that malarial parasites incorporated human PRDX2 for peroxide detoxification [45], suggesting that ART may also exert its anti-malarial function through the inhibition of PRDXs represented by human PRDX2. Considering the critical role of the redox system in the physiology of the cell and the potent therapeutic activities of ART in various diseases, the identification of PRDXs as the targets of ART and, more generally, of other upcoming drugs would open new perspectives for the future drug development of other diseases.

Collectively, the presented data provide compelling evidence that ART exacerbates ROS production by directly binding to PRDX1 and PRDX2 and inhibiting their antioxidant activities, thereby triggering ferroptosis, a programmed cell death mechanism. Furthermore, ART exhibits selective cytotoxicity towards DLBCL cells without impacting normal cells and demonstrates an improvement in liver function in vivo. These findings provide robust support for the further development of ART as a novel and promising therapeutic agent for the treatment of refractory or relapsed DLBCL.

## Materials and methods

### Reagents and materials

Customized Biotin-ART (Biotin-ART) was purchased from RuiXi Biological (Shanxi, China). Cell Counting Kit-8 (CCK-8) was purchased from Yeasen (Shanghai, China). Ferrostatin-1 was purchased from MedChemExpress (Shanghai, China). Antibodies include: PRDX1 (Proteintech; Cat. 15816-1-AP), PRDX2 (Proteintech; Cat. 10545-2-AP), PRDX3 (Proteintech; Cat. 10664-1-AP), GPX4 (Cell Signaling Technology, Cat. 52455 S), GAPDH (Proteintech; Cat. 10494-1-AP), and horseradish peroxidase-conjugated goat anti-rabbit/mouse secondary antibodies (Jackson ImmunoResearch, PA, USA).

### Cell culture

Human U2932, Riva, and OCI-Ly8 DLBCL cell lines were purchased from ATCC, American Type Culture Collection (Manassas, USA). Human GM12878 cell lines were purchased from BNCC (Zhejiang, China). These cells were kept in a 5% CO_2_ incubator, humidified in Dulbecco Modified Eagle Medium (DMEM) (Life Technologies, Gaithersburg, MD) containing 10% fetal bovine serum (FBS) (Life Technologies) at 37 °C.

### Detection of cell viability

Cells were seeded in a 96-well plate at a density of 2 × 10^3^ cells/well overnight, and then incubated with different concentrations of drugs for 24 or 72 h. CCK-8 kit was used to measure the viability of treated cells according to the manufacturer’s instructions.

### Determination of reactive oxygen species (ROS) levels

CM-H_2_DCFDA (GLPBIO, USA) was used for the detection of total ROS levels [46]. In short, the cells were collected, gently washed, resuspended in 1 mL phosphate-buffered saline (PBS), and then incubated in 10 μM H_2_DCFDA at 37 °C for 60 min. The cells labeled with fluorescent probes were centrifuged, the supernatant was discarded, and then resuspended. Flow cytometry was used to analyse fluorescent signals.

### Determination of lipid peroxidation

Lipid ROS levels were determined using BODIPY 581/591C11 (GLPBIO, USA), as per the manufacturer’s instructions [47]. Malondialdehyde (MDA) content was quantified using a lipid peroxidation MDA assay kit (Beyotime, Shanghai, China) and a Multi-Mode Microplate Reader (BioTek, Vermont, USA).

### Western blotting

The protein expression in the cells was analyzed by western blotting as described previously [8]. Cell cultures were lysed and centrifuged to collect supernatants. The protein amounts were determined using a BCA kit (Beyotime). Proteins were then separated by 12% SDS-PAGE and transferred to polyvinylidene difluoride (PVDF) membranes (EMD Millipore, MA, USA). Subsequently, the membranes were blocked in 5% dry skimmed milk at room temperature (RT) for 1 h, incubated with the primary antibodies at 4 °C overnight, and then with secondary antibodies at RT for 1 h. Proteins were detected using an enhanced chemiluminescence kit (Beyotime).

### Pull-down assay and liquid chromatography tandem-mass spectrometry (LC–MS/MS)

Soluble proteins from U2932 cells were extracted and incubated with Biotin-ART (100 μM) or biotin-DMSO (100 μM) for 4 h. Then, 100 μL of streptavidin-coated magnetic beads are added for incubation at 4 °C overnight. The beads were then washed several times and isolated using magnetic force, and the Biotin-ART-bound proteins were proceeded to LC–MS/MS analysis for identification.

For target identification by LC–MS/MS, the enriched proteins from streptavidin beads were separated by SDS-PAGE, followed by Coomassie staining. The band corresponding to the specific molecular weight region was excised, cut into small pieces, and then washed. After dehydration in 100% acetonitrile, the samples were reduced by dithiothreitol (DTT) and alkylated by iodoacetamide (IAA). Then the samples were incubated with trypsin to be digested into peptides overnight at 37 °C. The peptide solution was desalted on a C18 column. Finally, samples were analyzed by LC–MS/MS (Thermo).

For the confirmation of ART with PRDX1 and PRDX2, Biotin-ART pull down was similarly carried out, followed by Western blotting analysis using appropriate antibodies.

### Thermal shift assay (CETSA)

The Cellular Thermal Shift Assay (CETSA) has become a widely used method for studying ligand-protein interactions in mammalian cells. In this assay, drug-treated cells undergo heat denaturation, lysis, and centrifugation, during which ligand-bound proteins remain soluble in the supernatant while unbound proteins precipitate. The stabilized target proteins are then quantified by western blotting, providing direct evidence of drug-protein interactions under physiological conditions. In our experiments, U2932 cell lysates were incubated with either ART or DMSO control for 30 min at room temperature. Protein aliquots were then heated at graded temperatures (37–75 °C), centrifuged, and the soluble fractions were analyzed by Western blotting.

### Plasmids and protein purification

Human PRDX1 (GenBank ID:5052), PRDX2 (GenBank ID:7001), single-point mutated PRDX1 (G4A and G117A), and mutated PRDX2 (G4A, R7H, and T120S) were subcloned into the pET28a vector to generate wild-type and mutated proteins. Wild-type and mutated pET28a-PRDX1 plasmids were transformed into *Escherichia coli* BL21, and the protein expression was induced with 0.4 mM isopropyl-d-1-thiogalactoside. Wild-type and mutated pET28a-PRDX2 plasmids were transformed into *Escherichia coli* Rosetta, and the protein was expressed by inducing with 0.1 mM isopropyl-d-1-thiogalactoside. Then, the recombinant human PRDX1 protein (rhPRDX1) and recombinant human PRDX2 protein (rhPRDX2) were extracted and purified using the His protein purification kit (Beyotime). The purity and integrity of the recombinant protein were verified using Coomassie Brilliant Blue staining.

### Fluorescence spectrometry

rhPRDXs-ART binding was detected using a fluorescence spectrometer (F7000, Hitachi, Japan). 1 μM solution of rhPRDX1 or rhPRDX2, and 3 mM solution of ART was prepared using 20 mM Tris-HCl (pH 7.4, containing 10 mM NaCl). The parameters were set as follows: the excitation wavelength was 280 nm, the data were captured from 290 to 400 nm at RT, and the slit width of excitation was 5 nm and that of the emission beam was 5 nm. ART was gradually added into 1 mM rhPRDX1/rhPRDX2 until the fluorescence intensity was stable, and the fluorescence spectrum of rhPRDX1/rhPRDX2 at 290~400 nm was recorded. Each scan was repeated three times, and the titration curves were analyzed in Origin version 10.1, and the binding affinity (Ka) was calculated using Hill plots.

### Molecular docking

The crystal structures of proteins were retrieved from the Protein Data Bank (PRDX1: 4XCS; PRDX2: 5IJT). Docking process: Discovery Studio Client was used to perform dehydration and hydrogenation of proteins. AutoDock Vina 39 was used for molecular docking, and Pymol software for mapping.

### Circular dichroism (CD) spectroscopy

For CD experiments, rhPRDXs were dialyzed into 20 mM Tris-HCl buffer with the final concentration of 10 µM. CD spectra were recorded for rhPRDXs in the absence or presence of 1 mM ART at RT using an Applied Photophysics Chirascan spectrometry with a 0.1 cm pathlength quartz cuvette. For secondary structure characterization, CD spectra were recorded from 190 to 260 nm in stepwise of 1 nm at a scan rate of 120 nm/min. Three scans were averaged for each spectrum, and the spectrum of the buffer as the reference was subtracted. Estimations of the protein secondary structures were made using the CDNN software package.

### Activity assay of recombinant human PRDX proteins

rhPRDX1/2 proteins (20 μM) were incubated with different concentrations of DMSO or ART for 30 min, and added 10 μL H_2_O_2_ into different concentrations of solutions for 30 min at RT, followed by assessing the residual H_2_O_2_ level using Hydrogen Peroxide Assay Kit (Beyotime).

### Transfection assay

Complementary oligonucleotide sequences of shRNAs were designed and synthesized by IGE (Guangzhou, China). These shRNA or negative control (NC) vectors were transfected into human embryonic kidney 293 cells by Lipofectamine 2000.

### In vivo animal experiments

Male BALB/c-Nude mice (age, 5 weeks; body weight, 22 ± 2 g) were purchased from Guangdong Yaokang Biotechnology Co., Ltd., and maintained under standard conditions. After a week of adaptation to their surroundings, the mice were subcutaneously injected with 5 × 10^6^ U2932 cells into the right flank near the hind leg of each mouse. Following the growth of palpable tumors, the mice were randomly divided into two groups (*n* = 6 mice/group) and treated with 100 μl PBS or ART (100 μM) *via* intragastric injection. The tumor volume was measured every day and calculated using the following formula: volume = 1/2 (length × width^2^). The body weights were recorded once every 3 days. 12 days later, all mice were anesthetized to collect serum and liver, heart samples, and the tumors were harvested and weighed. The animal experimental protocol was approved by the Ethics Committee of Jinan University (Guangzhou, China).

### Biochemical and histological assays

Alanine aminotransferase (ALT) and aspartate transaminase (AST) levels were detected by Nuclee Biotechnology Co., Ltd. (Guangzhou, China). Liver and heart tissues were fixed, embedded, sectioned, stained with hematoxylin and eosin (H&E) for histological examination, and stained with Sirius red and Masson. Liver and tissue samples were examined under a light microscope (ECLIPSE Ts2R, Nikon, Japan) for each microscopic field.

### Statistical analysis

All experiments were repeated in triplicate in three independent experiments. Two-tailed Student’s *t*-test was used to analyze the significant differences between the two groups, and two-way analysis of variance (ANOVA) with Dunnett’s post hoc test, using GraphPad Prism 8 software (GraphPad Software. Inc., San Diego, CA, USA) was used to analyse significant differences between the multiple groups. *p*-values < 0.05 were considered statistically significant.

## Supplementary information

Supplementary Figures and their corresponding legends

Table S1 The proteins with differential expression by Bio-ART treatment

Table S2 shRNA lentivirus for PRDX1 and PRDX2

The uncropped images of all the main blots

## Data Availability

All data generated and analyzed during this study are included in this article and its Supplementary files. The data and materials used in this study are available upon reasonable requests to the corresponding author.
