# Correction: Artesunate induces ferroptosis in diffuse large B-cell lymphoma cells by targeting PRDX1 and PRDX2

**DOI:** 10.1038/s41419-026-08776-0

**Published:** 2026-04-29

**Authors:** Xiaohui Liu, Liyi Zeng, Jing Liu, Yulun Huang, Hua Yao, Jinman Zhong, Jiewen Tan, Xuejuan Gao, Dan Xiong, Langxia Liu

**Affiliations:** 1 MOE Key Laboratory of Tumor Molecular Biology and Key Laboratory of Functional Protein Research of Guangdong Higher Education Institutes, Institute of Life and Health Engineering, Jinan University, Guangzhou, China; 2 Department of Hematology, Shunde Hospital, Southern Medical University (The First People’s Hospital of Shunde), Foshan, Guangdong China

**Keywords:** Mechanism of action, Drug development, Drug regulation, Targeted therapies, Cell death

Correction to: *Cell Death & Disease* 10.1038/s41419-025-07822-7, published online 11 July 2025

The term “Xuenjuan Gao” in the original text is confirmed to be a typing error. The correct name of the corresponding author is “Xuejuan Gao”—the redundant letter “n” in “Xuenjuan” is an unintended typo that deviates from the author’s official and intended name.

Since author name accuracy is critical for academic attribution, citation integrity, and professional recognition, this correction is essential to avoid misidentification of the corresponding author, ensure proper academic credit, and maintain the document’s professionalism. The revision aligns with standard academic writing practices, eliminating potential confusion for readers, researchers, and citation databases while upholding the integrity of the author information.

The original article has been corrected.

