# Supplementary material for: Artesunate induces ferroptosis in diffuse large B-cell lymphoma cells by targeting PRDX1 and PRDX2
Source: Cell Death Dis. 2025 Jul 11;16(1):513. doi: 10.1038/s41419-025-07822-7 (PMC12254379; doi:10.1038/s41419-025-07822-7)
Supplement: Supplementary file 1 — Supplementary Figures and their corresponding legends [file 41419_2025_7822_MOESM1_ESM.docx]

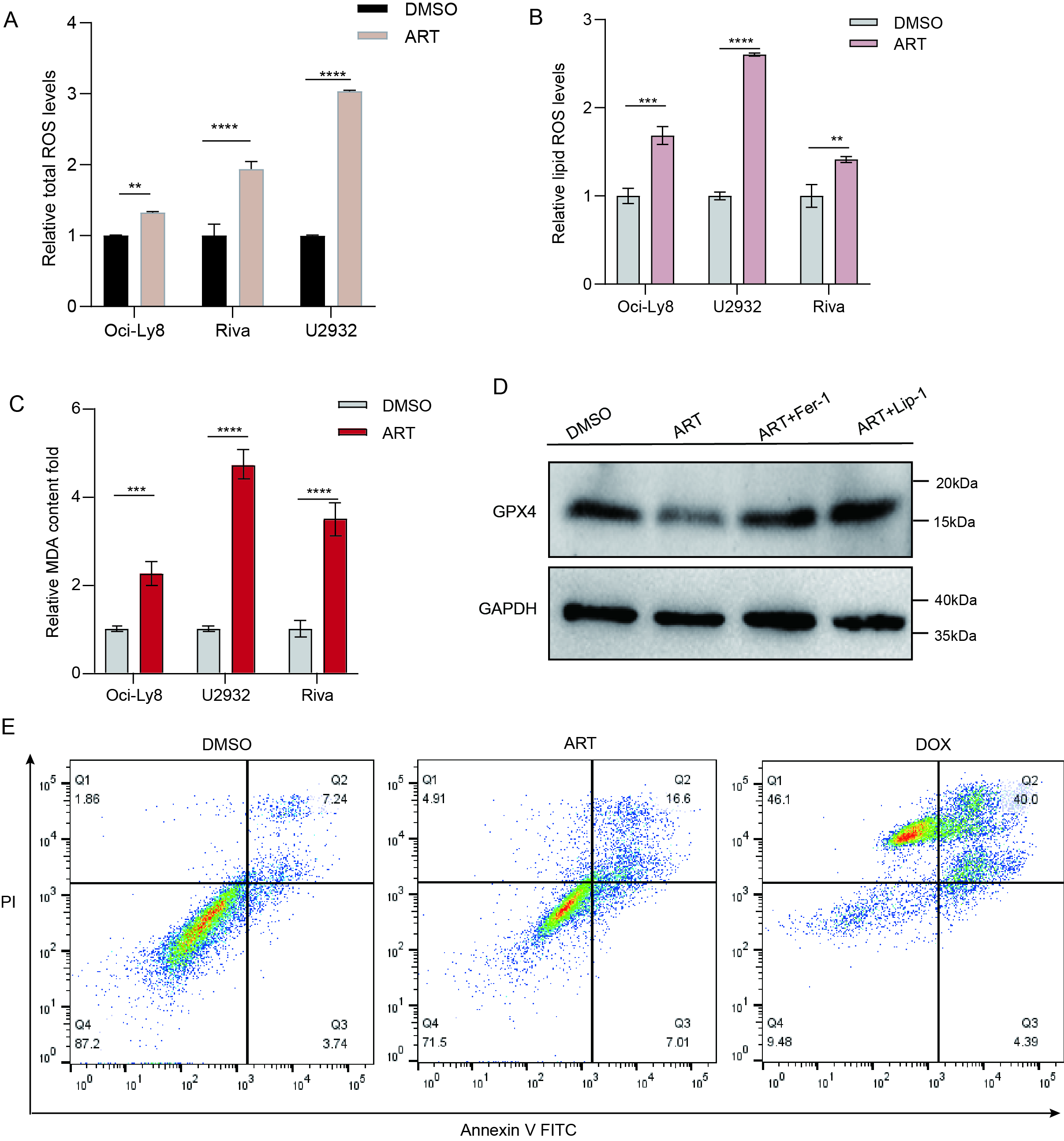


**Supplementary Fig. 1 ART induces cell death via ferroptosis and apoptosis**

U2932, Riva and Oci-Ly8 cells pretreated with or without ART (100 µM) for 24 h were labeled with a 10 µM DCFH-DA (**A**) or 10 µM C11 BODIPY (**B**) fluorescent probe, respectively, and total ROS or lipid ROS levels were detected by flow cytometry. Detection of cellular MDA levels (**C**) in U2932, Riva and Oci-Ly8 cells treated with or without ART (100 µM) for 24 h. (**D**) U2932 cells were treated with or without ART (100 µM) in the presence or absence of pretreatment with different inhibitors for 24 h. These inhibitors included Ferrostatin-1 (Fer-1, an inhibitor of ferroptosis, 50 μM), liproxstatin-1 (Lip-1, an inhibitor of lipid peroxidation, 1µM) for 24 h, then the protein expression levels of GPX4 were assayed. (**E**) Annexin V-FITC/PI staining assay of apoptotic U2932 cells after treated with ART (100 μM), or doxorubicin (DOX, 10 μM) for 24 h. Data are presented as mean ± s.d. from three independent experiments. **: *p*<0.01，***: *p*<0.001, ****: *p* < 0.0001, determined by two-way ANOVA.


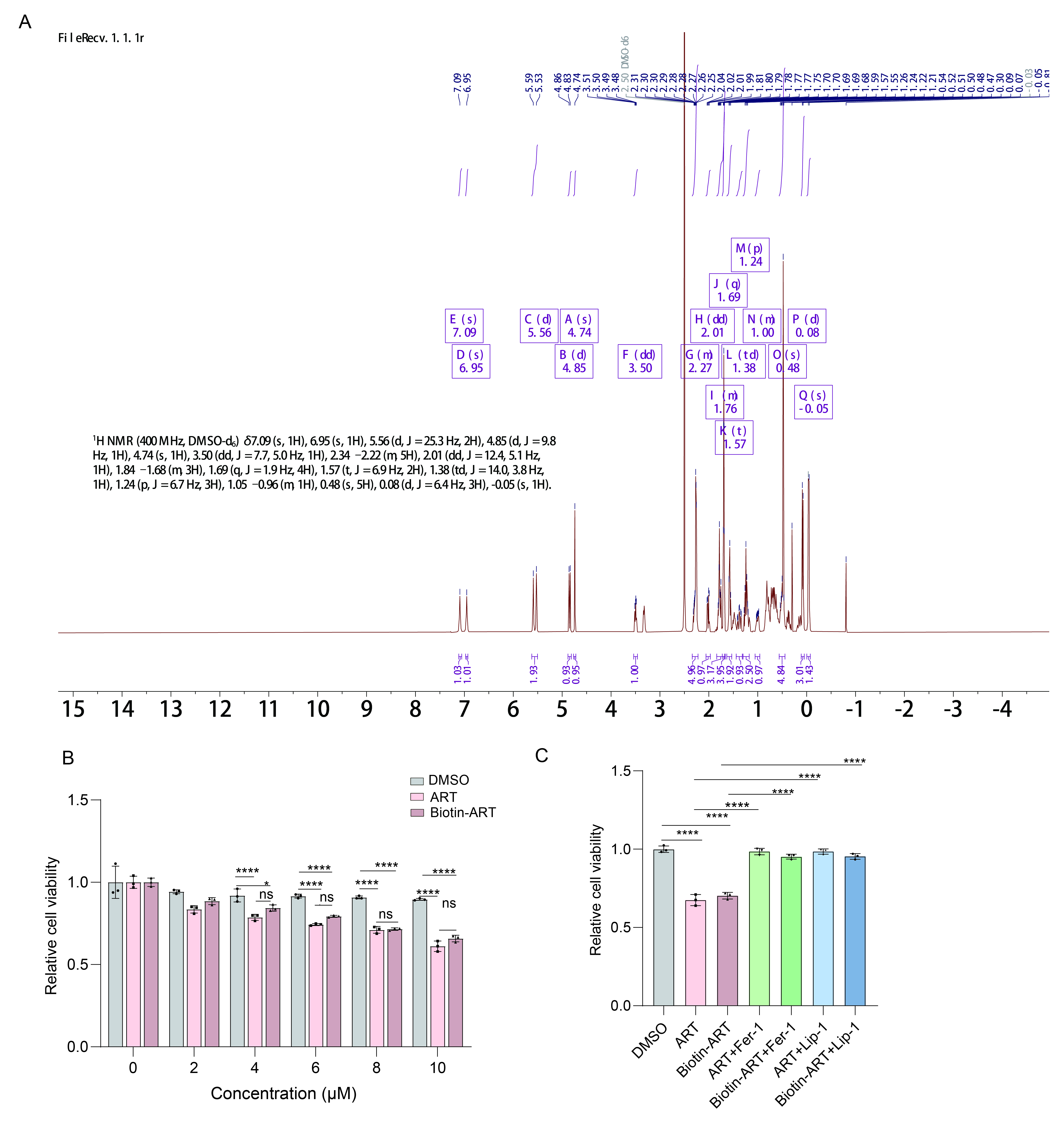


**Supplementary Fig. 2 Identification of ART target proteins using SM-Pull down and LC-MS/MS**

(**A**)The structure of Biotin-ART was validated using high-resolution nuclear magnetic resonance (HNMR). (**B**)The effect of ART and Biotin-ART under the indicated gradient concentration was determined by CCK8 assay. (**C**) U2932 cells were treated with ART (10 μM), or Biotin-ART (10 μM) in the presence or absence of pretreatment with different inhibitors for 24 h. These inhibitors included Fer-1(50 μM), Lip-1(1 µM). The cell viability was measured by CCK8 assay. Data are presented as mean ± s.d. from three independent experiments. ****: *p* < 0.0001, determined by two-way ANOVA.


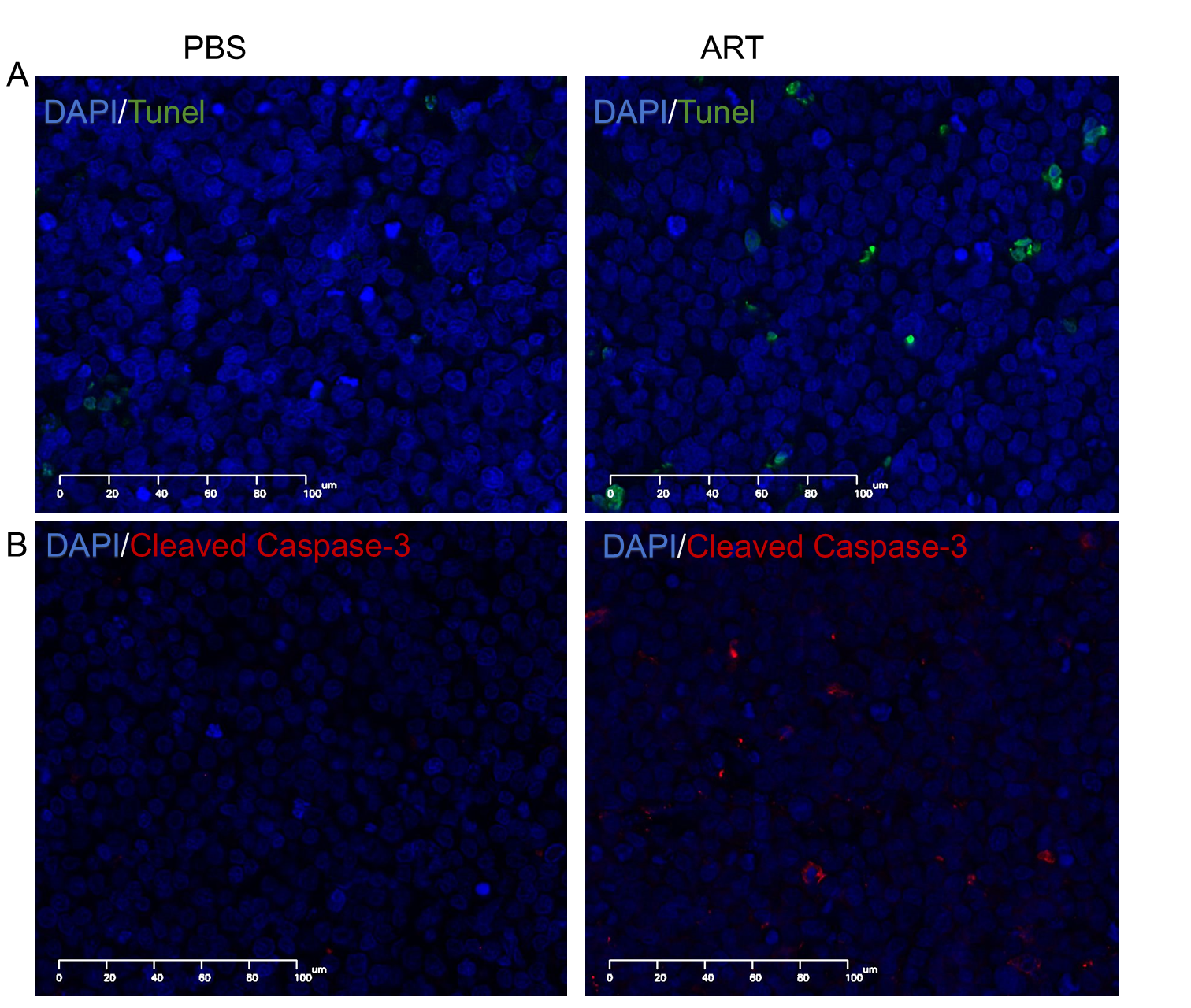
**Supplementary Fig. 3 ART induces moderate pro-apoptotic effects *in vivo***

TUNEL staining (**A**) and cleaved caspase-3 immunofluorescence (**B**) in tumor tissues.
