# Supplementary material for: Artesunate induces ferroptosis in diffuse large B-cell lymphoma cells by targeting PRDX1 and PRDX2
Source: Cell Death Dis. 2025 Jul 11;16(1):513. doi: 10.1038/s41419-025-07822-7 (PMC12254379; doi:10.1038/s41419-025-07822-7)
Supplement: Supplementary file 3 — Table S2 shRNA lentivirus for PRDX1 and PRDX2 [file 41419_2025_7822_MOESM3_ESM.docx]

| Name | Sequence |
| --- | --- |
| control shRNA | 5’-UUUGUACUACACAAAAGUACUG-3’ |
| shPRDX1-1 | 5’-CCTTCGCCAGATCACTGTTAA-3’ |
| shPRDX1-2 | 5’-GCCTGGCAGTGACACGATTAA-3’ |
| shPRDX1-3 | 5’-CCAGATGGTCAGTTTAAACAT-3’ |
| shPRDX2-1 | 5’-CCTTCGCCAGATCACTGTTAA-3’ |
| shPRDX2-2 | 5’-GCCTGGCAGTGACACGATTAA-3’ |
| shPRDX2-3 | 5’-GTGAAGCTGTCGGACTACAAA-3’ |

**Table S2 shRNA lentivirus for PRDX1 and PRDX2**
