# Supplementary figures and images for: Artesunate induces ferroptosis in diffuse large B-cell lymphoma cells by targeting PRDX1 and PRDX2
Source: Cell Death Dis. 2025 Jul 11;16(1):513. doi: 10.1038/s41419-025-07822-7 (PMC12254379; doi:10.1038/s41419-025-07822-7)

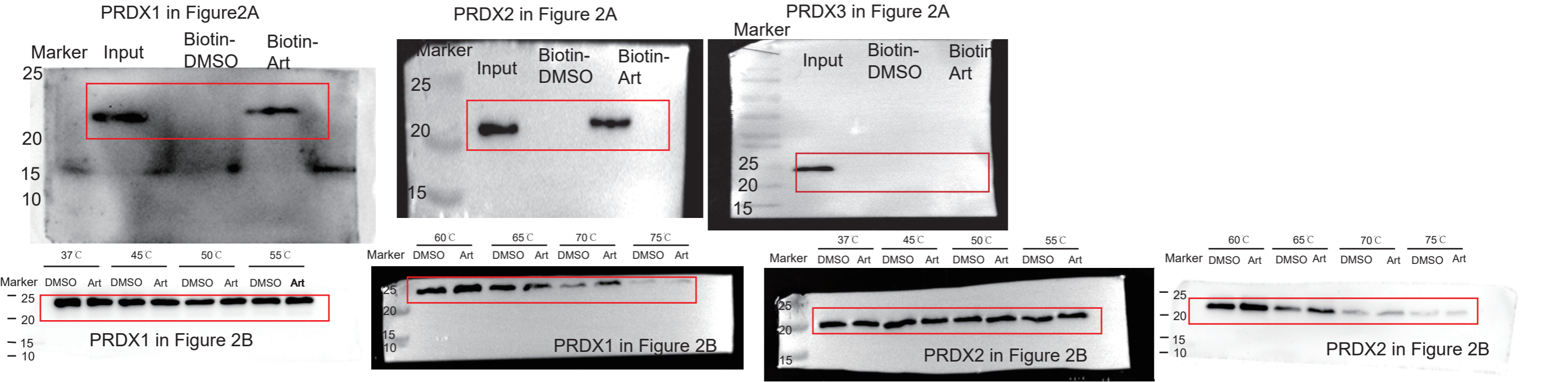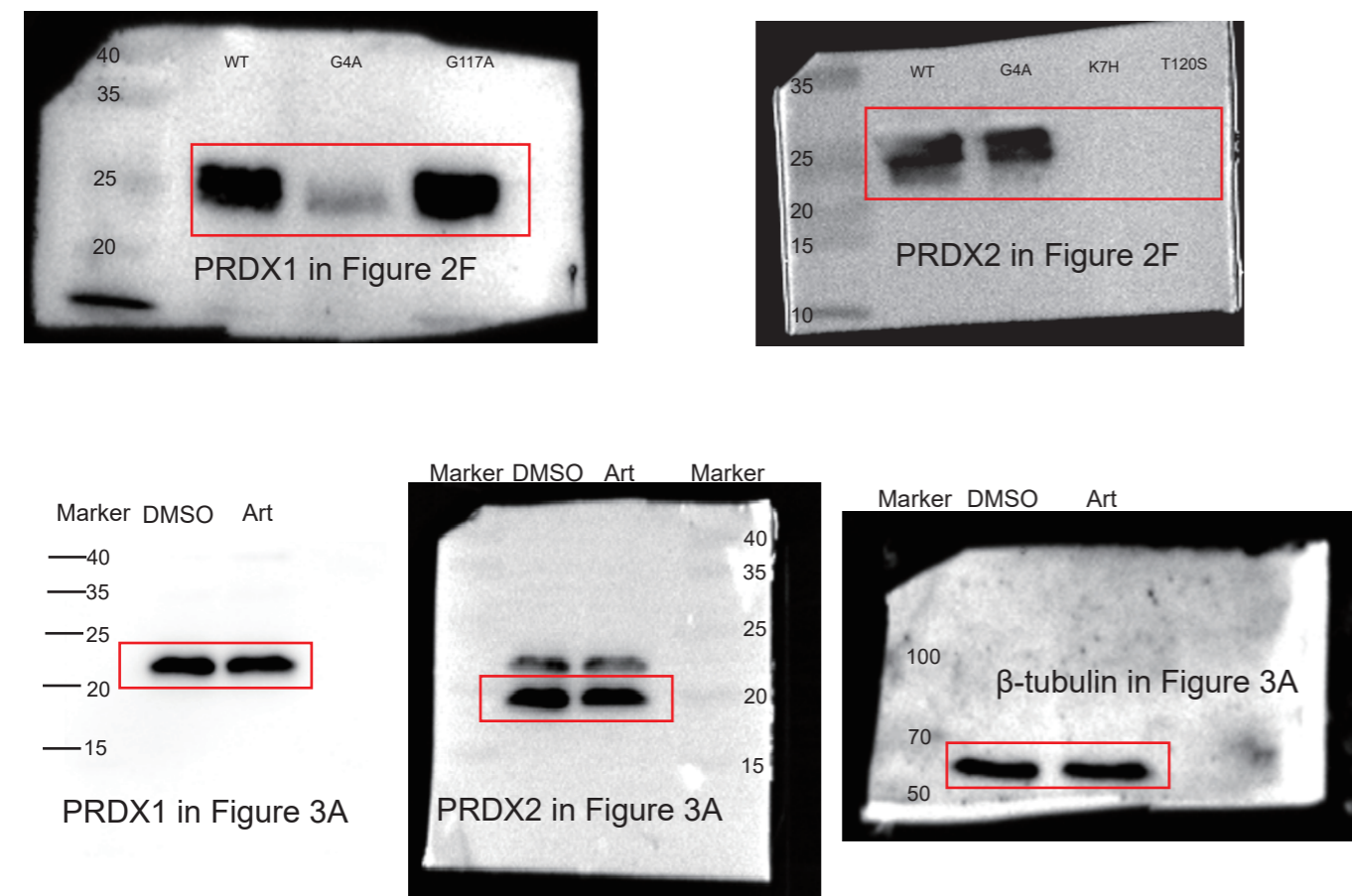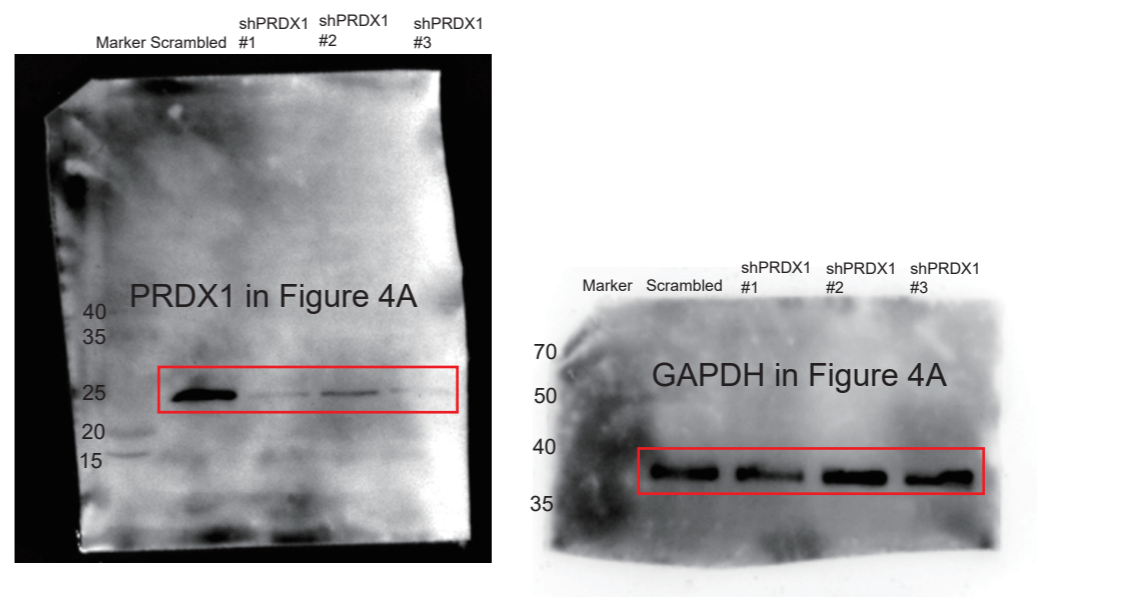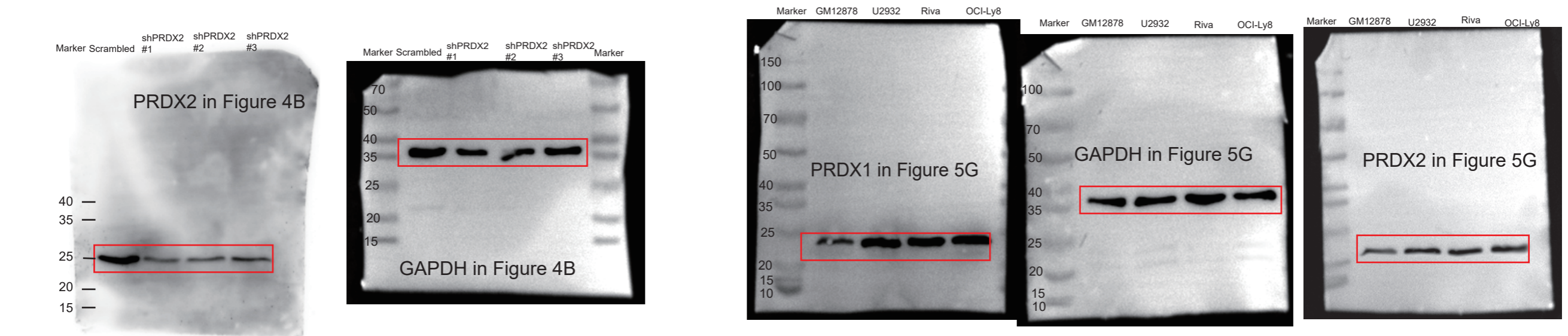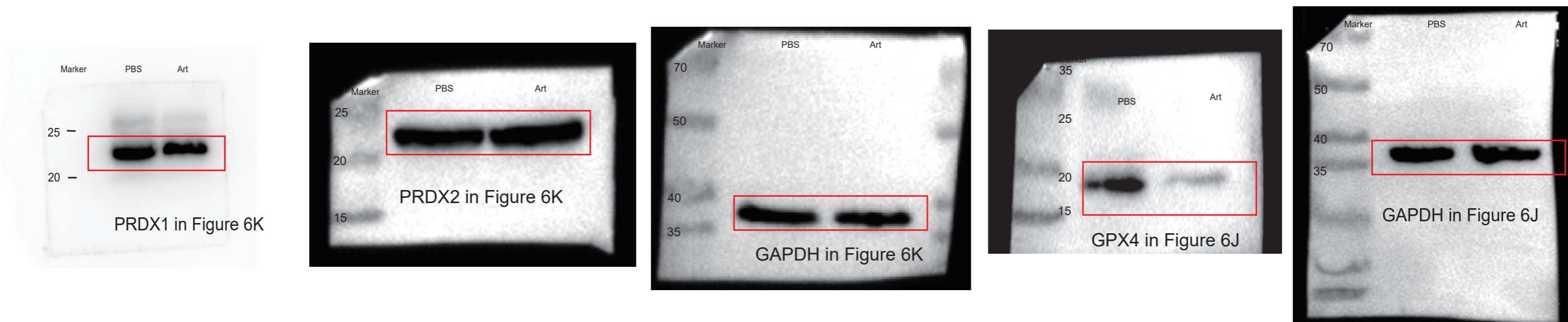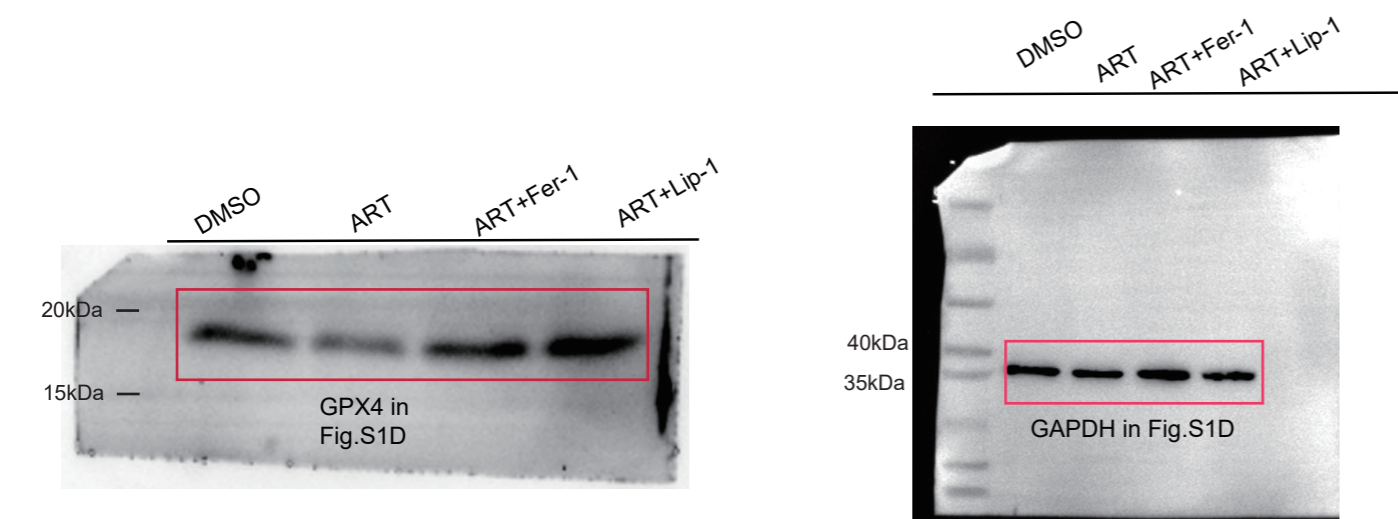

Supplement: Supplementary file 4 — The uncropped images of all the main blots [file 41419_2025_7822_MOESM4_ESM.pdf]
